# Monitoring *Candida parapsilosis* and *Staphylococcus epidermidis* Biofilms by a Combination of Scanning Electron Microscopy and Raman Spectroscopy

**DOI:** 10.3390/s18124089

**Published:** 2018-11-22

**Authors:** Kamila Hrubanova, Vladislav Krzyzanek, Jana Nebesarova, Filip Ruzicka, Zdenek Pilat, Ota Samek

**Affiliations:** 1 Institute of Scientific Instruments of the Czech Academy of Sciences, CZ-61264 Brno, Czech Republic; hrubanova@isibrno.cz (K.H.); krzyzanek@isibrno.cz (V.K.); pilat@isibrno.cz (Z.P.); 2 Biology Centre of the Czech Academy of Sciences, CZ-37005 Ceske Budejovice, Czech Republic; nebe@paru.cas.cz; 3 Department of Microbiology, Faculty of Medicine, Masaryk University and St. Anne’s Faculty Hospital, CZ-65691 Brno, Czech Republic; fruzic@fnusa.cz

**Keywords:** Raman spectroscopy, biofilm, sample preparation, scanning electron microscopy, cryo-SEM

## Abstract

The biofilm-forming microbial species *Candida parapsilosis* and *Staphylococcus epidermidis* have been recently linked to serious infections associated with implanted medical devices. We studied microbial biofilms by high resolution scanning electron microscopy (SEM), which allowed us to visualize the biofilm structure, including the distribution of cells inside the extracellular matrix and the areas of surface adhesion. We compared classical SEM (chemically fixed samples) with cryogenic SEM, which employs physical sample preparation based on plunging the sample into various liquid cryogens, as well as high-pressure freezing (HPF). For imaging the biofilm interior, we applied the freeze-fracture technique. In this study, we show that the different means of sample preparation have a fundamental influence on the observed biofilm structure. We complemented the SEM observations with Raman spectroscopic analysis, which allowed us to assess the time-dependent chemical composition changes of the biofilm in vivo. We identified the individual spectral peaks of the biomolecules present in the biofilm and we employed principal component analysis (PCA) to follow the temporal development of the chemical composition.

## 1. Introduction

Yeast and bacteria are microorganisms that can live as planktonic cells or in an organized formation called biofilm [1]. During their adherence to surfaces or interfaces and their subsequent proliferation, the cells embed themselves into an amorphous extracellular matrix (ECM) [2], which is composed of extracellular polymeric substances (EPS) produced by the cells [3,4]. The presence of ECM is often considered an important characteristic of a mature biofilm [5]. Among the main components of EPS is a mixture of polysaccharides, proteins, and extracellular DNA [6,7]. Since these substances are all highly hydrophilic, the biofilm water content can sometimes be as high as 90% of the total biofilm mass [1,8]. 

Microbes in natural ecosystems appear to have a pronounced tendency to colonize various surfaces and each other. The interest in such microbial communities—biofilms—has increased over the last three decades, because the biofilms are important in many aspects of health, biotechnology, etc. [9]. The presence of biofilms can lead to human health problems, e.g., biofilm on teeth [10], also known as plaque and a factor in tooth decay and parodontosis, as well as the development of biofilm on medical devices such as catheters or implants [11]. On the other hand, biofilms can be useful, as they are already extensively used in wastewater treatment [12,13] and play a role in biofuel production such as methane generation by methanogenesis [14] or in food production [15]. Life in biofilms is favorable for microbes, bringing advantages such as enhanced persistence and resistance to environmental threats such as antimicrobial agents [16], toxic substances, thermal and oxidative stress [2,17]. Although the composition of biofilms varies depending on the system under study, in general the major component of a biofilm is water. Apart from water and the bacterial cells, the biofilm matrix is a complex formed principally by exopolysaccharides [18]. Moreover, other macromolecules such as proteins, DNA and various products from the lysis of bacteria are present in the biofilm matrix [19]. Studies of biofilms suggest that the biofilm matrix architecture is variable and it contains channels that enable water, nutrients and oxygen flow through the biofilm [20]. However, the detailed architecture of the channels inside the ECM, and the processes operating within them have not yet been fully elucidated [16,20]. Therefore, in this study we have combined suitable microscopic and spectroscopic techniques that could be useful for studying the biofilms.

We examined the biofilm with emphasis on the differences in the apparent structure of the ECM, linked to various sample preparation protocols for SEM. The yeast *Candida parapsilosis* and bacterium *Staphylococcus epidermidis* have been studied in this project. These species are frequently found among the normal human microbiota [21,22]. However, in a medical context, the ability to form biofilms allows these microbes to colonize the surfaces of implants, consequently causing difficult-to-treat infections, especially in immunocompromised patients [23,24]. The presence of EPS protects the microbial cells from the natural defenses of the human immune system as well as from the effects of antibiotic treatments [25,26] and thus it complicates the therapy [1,8]. Understanding the biofilm structure can contribute to the research of biofilm formation and the underlying biochemical mechanisms. This will help to develop a more efficient treatment strategy for biofilm infections [27,28]. 

Microbial biofilms are usually investigated by various microscopic techniques including confocal laser scanning microscopy (CLSM) and conventional scanning electron microscopy (SEM) [13,29,30], transmission electron microscopy (TEM) [31], Focused Ion Beam (FIB)-SEM [3] and by special SEM techniques, such as cryo-SEM or environmental-SEM [3,32,33,34]. The main limitation of the light microscopy techniques is the restricted magnification [35]. This can be resolved by the use of SEM, which provides high-magnification images of the individual bacteria and yeast cells and their location and interaction within the ECM, which is important for understanding the morphology and physiology of biofilms [2,36]. However, a conventional SEM, where the sample is observed in high vacuum at room temperature, is limited due to the need for a dry sample [37]. Biofilms are rich in water and the conventional sample preparation for SEM that includes desiccation as a prerequisite for imaging can cause substantial changes in the ECM and the microbial cell ultrastructure, leading to artifacts [5,34]. Chemical fixation with aldehydes and osmium tetroxide treatment help to preserve cell morphology and enhance contrast [38,39], while dehydration with ethanol or acetone series is used for the gradual replacement of the water inside the sample. However, this mode of preparation also causes some artifacts, such as cell membrane discontinuities [40], and it has other deleterious effects on morphology. In the case of cryo-fixation, the biofilm is not dehydrated but kept frozen to obtain high-resolution images closer to the native state of the sample [37,39,41]. It has been proven that in cryo-fixed biofilms, the bacterial ultrastructure preservation and the biofilm organization improved significantly [42]. To reduce the damage inherent to these treatments, various innovative cryogenic sample preparation methods have been developed [41,43,44]. One of the simplest cryo-fixation techniques is plunging the sample into a liquid cryogen [45]. In general, plunging into liquid nitrogen is not usually sufficient because of the Leidenfrost effect: a thermally insulating film of vaporized nitrogen forming around the sample, preventing fast cooling and allowing water ice crystals to form inside the specimen [46]. However, cryogens like liquid ethane/propane are often used, for example in electron tomography, for fixation of very thin layers [42]. Substantially more effective freezing can be achieved by increasing the pressure during exposure to the liquid cryogen. This can be performed by the high-pressure freezing (HPF) technique [47,48,49,50]. 

We coupled the SEM morphological examination of biofilms with chemical characterization by Raman microspectroscopy. Raman microspectroscopy employs a laser beam that is focused with the microscope objective lens in order to excite and collect Raman scattering from a small volume of the sample. Raman spectra from living microorganisms contain multiple spectral peaks corresponding to unique interatomic vibrations in biomolecules, e.g., nucleic acids, proteins, carbohydrates, and lipids [51,52,53,54]. It has been shown that Raman microspectroscopy and Raman imaging can be regarded as the methods of choice for many studies of microorganisms, cells and other biological samples [51,55,56,57,58,59,60,61,62,63,64,65]. Detailed databases of Raman spectral features encountered in biological samples had been published before [60]. When characterizing biofilms using Raman microspectroscopy, the common approach is to analyze the biofilm as a whole. The spectra can be acquired point-by-point at selected positions or using line-scan techniques such as Renishaw StreamLine. In such cases, the Raman signal originates from the cells as well as from the ECM. Nevertheless, it may be useful to separate the ECM contribution from the Raman spectra, in order to fully understand the biochemical processes in the cells embedded in the biofilm matrix. It is well known that such cells express phenotypes that differ from those of their planktonic counterparts, i.a. the increased resistance to chemical treatments. 

We employed SEM to study the ECM content and distribution in the biofilm, and the way it translates into its Raman spectral characteristics. The SEM images helped us to estimate the relative proportion of the ECM, which is in most cases ranges between 20% and 50% of the total biofilm volume. This means that in the Raman spectra, we observe the signal both from the bacterial cells and from the ECM, proportion of which depends on the growth stage of the biofilm. The proportion of the ECM increases with the age of the biofilm.

## 2. Materials and Methods

### 2.1. Biofilm Cultivation

Two biofilm-positive microbial strains that are often involved in serious infections [27,36,66] were selected as model organisms and examined in this study: the well-characterized *ica* operon-positive, biofilm and slime producing *Staphyloccocus epidermidis* strain CCM 7221 (Czech Collection of Microorganisms, Brno, Czech Republic) [4] and *Candida parapsilosis* BC11 from the Collection of the Microbiology Institute, Masaryk University and St. Anne’s University Hospital (Brno, Czech Republic) [66]. The strains included in this study were stored at −70 °C in cryo-tubes (ITEST plus, Hradec Králové, Czech Republic). Prior to each experiment, the strains were thawed quickly at 37 °C and cultivated on Mueller-Hinton agar (Oxoid, Basingstoke, UK) at 37 °C for 24 h. The microbial cultures were re-suspended in a sterile physiological saline solution (PSS) to the optical density 0.5 of the McFarland scale [54]. 

In our experiments with the yeast biofilm, the wells of 24-well polystyrene tissue culture plates Nunclon (Nunc, Roskilde, Denmark) containing 1 mL of Yeast Nitrogen Base medium Difco (Becton, Dickinson and Co., Franklin Lakes, NJ, USA) with 4% glucose (YNBg) and sterile substrate discs were inoculated with 100 μL of standardized cell suspension. Bacterial cultures were cultivated in 1 mL of brain-heart infusion (BHI) medium (Oxoid) with 4% glucose (BHIg) under the same conditions. We used standard cover slips or sapphire discs with the diameter 1.4 mm (No. 16706849, Leica Microsystems, Vienna, Austria) and 6 mm (No. 16770158, Leica Microsystems) as a substrate for HPF freezing and a cover slip (No. 1014/1818, Hecht-Assistant, Paris, France) for plunge freezing and conventional protocols for SEM; cover slips are widely used as a cultivation substrate for in vitro biofilm experiments [67,68,69]. After 24 h of incubation at 37 °C the substrate discs were removed from wells and further processed.

### 2.2. Conventional SEM

**Figure 1:**
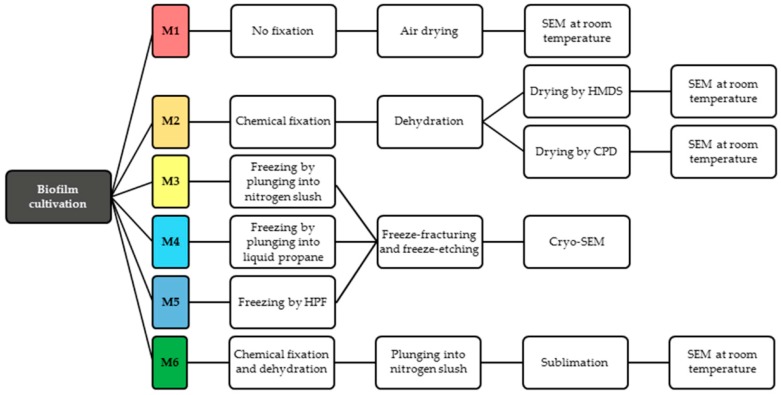
Diagram of microbial biofilm sample preparation for SEM.

All the samples were imaged by several different high vacuum scanning electron microscopes (SEM) at room temperature. Specifications of the SEM devices and information on the imaging parameters are stated along with each procedure below in the text. Figure 1 summarizes all the sample preparation protocols for conventional SEM used in our experiments.

#### 2.2.1. Method 1—Air-Drying (M1)

The simplest way to visualize the biofilm in vacuum of electron microscope is without any fixation of the structure. The microbial cultures of *S. epidermidis* and *C. parapsilosis* were cultivated in a medium for 24 h on a cover glass. Subsequently, the samples were air dried for 30 min. Imaging of the SE signal was performed by a VEGA TS 5130MM scanning electron microscope (SEM) (Tescan Orsay Holding, Brno, Czech Republic) at the acceleration voltage of 10 kV with the use of a homemade cathode lens with the deceleration voltage in the range around 3 kV and the working distance of 10 mm.

#### 2.2.2. Method 2—Chemical Preparation (M2)

The microbial cultures of *S. epidermidis* and *C. parapsilosis* after 24-h cultivation under the same conditions as described in previous experiments were prepared according to the standard protocol [37,70,71]. Our samples were fixed in 2.5% glutaraldehyde (Sigma-Aldrich, St. Louis, MO, USA), post-fixed in 1% osmium tetroxide (OsO_4_; Sigma-Aldrich) and thoroughly but carefully washed by PBS buffer (Sigma-Aldrich). The process of dehydration by ethanol (VWR Chemicals, Leuven, Belgium) series (30%, 50%, 70%, 80%, 90%, 95%, each step 15 min, and three times 100%) prepared the samples for drying. Here two methods of drying are compared. The first was done by hexamethyldisilazane (SPI-Chem, West Chester, PA, USA; HMDS; CAS 999-97-3) which was diluted with acetone (Sigma-Aldrich). Therefore, before applying this treatment it was necessary to replace the ethanol with acetone in four steps with increasing proportion of acetone (ratio ethanol/acetone 2:1; 1:1; 1:2; pure acetone) [37,70,71]. The second methodology for sample drying is the critical point drying (CPD) using CO_2_. The samples, prepared by conventional protocols [37,70] were coated by 10 nm of Au before imaging in a VEGA TS 5130MM SEM at the acceleration voltage 10 kV or in a Magellan 400L SEM (Thermo Fisher Scientific, Hillsboro, OR, USA) at 2 kV.

### 2.3. Cryo-SEM

Our experiments with imaging at low temperature by cryo-SEM show the comparison of the biofilm structure at the same cultivation conditions but with different preparation protocols. We tested multiple cryo-fixation techniques; the workflow is summarized in Figure 1.

#### 2.3.1. Method 3—Plunging into Nitrogen Slush (M3)

Freezing in the nitrogen slush was performed in the slushing station, a part of the ALTO 2500 cryo-preparation system (GATAN Inc., Pleasanton, CA, USA) [72,73]. The substrate for the cultivation of the biofilm samples was a cover glass of thickness 0.17 mm that was removed from the medium without any rinsing immediately before the freezing step. After freezing, the sample was transferred into an ALTO 2500 cryo-preparation chamber, perpendicularly freeze-fractured and subjected to a short sublimation at −95 °C for 3 min. The imaging was performed by SEM 7401F (JEOL, Akishima, Japan). The cryo-stage in the SEM was cooled at −135 °C, the anti-contamination aperture at −145 °C during specimen observation. The secondary electron (SE) micrographs were recorded at an electron energy range between 1 and 2 keV (low dose), and the working distance of approximately 6–8 mm over the fracture plane.

#### 2.3.2. Method 4—Plunging into Liquid Ethane (M4)

Freezing in liquid ethane was performed in a homemade plunger. The substrate for cultivation of the biofilm samples was a cover glass of thickness 0.17 mm, which was removed from the medium without any rinsing immediately before the freezing step. After the freezing, the sample was mounted in a standard manner into a cryo-sample holder inside the nitrogen slush, then transferred into an ALTO 2500 cryo-preparation chamber, perpendicularly freeze-fractured and subjected to a short sublimation at −95 °C for 3 min. The imaging was performed by SEM 7401F (JEOL, Akishima, Japan) at the same conditions as described above.

#### 2.3.3. Method 5—Cryo-Preparation by HPF (M5)

The high pressure freezing was performed by the HPF instrument EM PACT2 (Leica Microsystems) in standard conditions according to the instructions given in the operation manual. With the HPF EM PACT2, it is only possible to freeze sapphire disks with a diameter of 1.4 mm and therefore they were used as the substrate for the biofilm cultivation. Just as in the case of the plunging fixation the samples were not rinsed before freezing. After freezing, the sample was mounted into a homemade cryo-sample holder in liquid nitrogen [34], then transferred into a cryo-preparation chamber ALTO 2500, perpendicularly freeze-fractured and subjected to short sublimation at −95 °C for 3 min. Imaging was performed by SEM 7401F (JEOL) under the same conditions as described above. Our experiment with high-pressure freezing of the biofilm of *Staphylococcus epidermidis* was carried out by means of a HPF EM ICE (Leica Microsystems). In this case we used 6 mm sapphire discs as a cultivation substrate. After the freeze-fracturing in the ACE 600 cryo-preparation chamber (Leica Microsystems) the samples were sublimated for 5 min at −95 °C and then transferred by the VCT 100 shuttle (Leica Microsystems) into a Magellan 400L cryo-SEM (Thermo Fisher Scientific). The fractured structures were observed with a 2 keV electron beam at −120 °C and a working distance of around 7 mm.

### 2.4. Method 6—Combined Preparation: Chemical and Cryo-Methods (M6)

Our experiments with combined sample preparation started with biofilm cultivation on the cover glass under the same cultivation conditions as described above, followed by chemical fixation by 2.5% glutaraldehyde and 1% OsO_4_ in PBS buffer and thorough washing with PBS. In the next step, the samples were dehydrated by ethanol series (30%, 50%, 70%, 80%, 90%, 95% *v*/*v* ethanol; 15 min each, and three times with 100% ethanol) and frozen by plunging into the nitrogen slush. After mounting into a standard cryo-sample holder, the samples were transferred into a vacuum chamber (ACE 600) where they were sublimated overnight. The samples were then moved under high vacuum using a shuttle (VCT 100) into the SEM (Magellan 400L) and observed with a 2 keV electron beam at room temperature. Working distance was around 6 mm. The samples were metal coated by 2 nm of Pt.

### 2.5. Analysis of the Yeast Biofilm by Raman Spectroscopy

We used a commercial Renishaw Raman microspectrometer (Renishaw inVia, Renishaw plc., Wotton-under-Edge, UK), with a 785 nm diode laser as the excitation source. The laser beam was focused on the sample with a microscope lens (Leica, Wetzlar, Germany, 50×, NA 0.5), the laser spot diameter was 2 µm × 10 µm (note that this laser spot shape is characteristic for the Renishaw InVia instrument [51,64,65]). 

The laser was focused on the surface of the biofilm, which was grown on CaF_2_ substrate. The samples were measured in two stages: first immediately after the cells were transferred to the substrate (no biofilm), and subsequently after 6 h of growth at 37 °C, when the fresh biofilm structures were formed. The spectra were measured for 30 s from different parts of the sample. The power of the excitation laser reached approximately 100 mW under the objective lens. The Raman spectra were treated with Savitzky-Golay filter to remove noise and with rolling circle filter for background fluorescence removal [74], and subsequently analyzed by principal component analysis [75]. The software was written using MatLab (MathWorks, Natick, MA, USA).

## 3. Results and Discussion

### 3.1. Conventional SEM (M1 and M2)

SEM was found suitable for examination of the microbial biofilms, allowing visualization of microbial cell surfaces and the surrounding ECM with high resolution. Conventional sample preparation protocols for fully hydrated biological material involve following primary steps such as chemical fixation, dehydration and drying. This process modifies the biological material for the low pressure in the SEM chamber during the imaging. The advantages of a microbial biofilm specimen prepared in this manner are its stability, easy manipulation at room temperature, and the possibility of additional coating, which helps to visualize details in the surface structure. Contrarily, the use of chemical treatments and multiple rinsing steps exacerbates the artifacts, especially in the sensitive ECM structure and some parts of the biofilm layer may be completely destroyed. 

**Figure 2:**
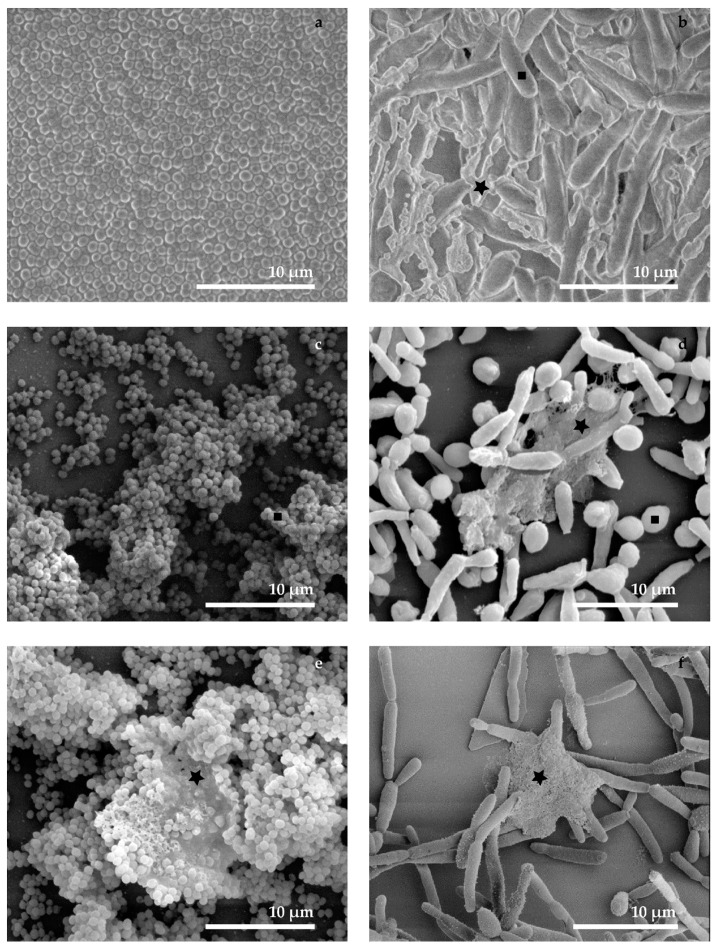
SEM micrographs show the comparison of preparation protocols for microbial biofilm structure (in the left column *S. epidermidis* and in the right column *C. parapsilosis*): (**a**,**b**) no chemical preparation and air drying; measurement parameters: 10 kV with the use of a homemade cathode lens with the deceleration voltage in the range around 3 kV, WD 10 mm; (**c**,**d**) chemical sample preparation and drying by CPD; measurement parameters: (**a**) 10 kV or (**b**) 2 kV, WD 8 mm; (**e**,**f**) chemical sample preparation and drying by HMDS. Marks: stars—ECM, squares—cells; measurement parameters: 2 kV, WD 8 mm.

The presented micrographs (Figure 2) show the comparison of influences of various preparation protocols on the microbial biofilm structure. The easiest way of biofilm preparation for SEM observation is air drying on a cover glass. In the micrographs (Figure 2a,b), the surface structures of the biofilm layer look similar for bacteria and yeast; the air drying caused a collapse of the three-dimensional structure. Moreover, in case of *Candida parapsilosis* we see that the extracellular space is filled by a dry matrix and the residues of the cultivation medium (Star in Figure 2b). It was not possible to visualize the clear surfaces of microbial cells (Square in Figure 2b) because they were covered by ECM and dry cultivation medium. 

The chemical fixation and dehydration by ethanol series allowed preservation of the three-dimensional structure of the biofilm. However, in many areas of the cultivation substrate, the biofilm layer was destroyed and washed away due to the rinsing process during the sample preparation. ECM was partially preserved as a compact and roughened fiber-like matter in the extracellular space (Figure 2c–f, stars). The use of HMDS as a drying solution is a gentler way than CPD, the biofilm layer was less perturbed. Furthermore, the choice of the drying technique following the chemical preparation did not influence ECM quality. Therefore, drying by HMDS is a preferable method of the biofilm preparation for room temperature imaging by SEM whenever cryo-methods cannot be applied. The conventional SEM can be used for observing the surface of bacterial and yeast biofilm and obtaining high-resolution images of the spatial distribution of microbes. It can clearly be seen that the sample preparation represents a crucial parameter in the preservation of the three-dimensional structure of the biofilm. The influence of the chemical treatment is most obvious on the spatial architecture of the biofilm and the compact structure of the ECM. The significant disadvantages of the chemical preparation are the partial loss of the biofilm from the cultivation substrate and the time requirements for this process.

### 3.2. Cryo-SEM (M3, M4 and M5)

The use of low-temperature preparation by cryo-SEM for observing biological samples brings a number of advantages compared to conventional SEM at room temperature. The samples do not have to be fixed by chemicals treatment and rinsed several times by a buffer. Moreover, the dehydration series is eliminated and the lengthy drying is no longer needed, therefore the artifacts associated with these processes are eliminated. On the other hand, the observation of biological structures in SEM at low temperatures requires specialized equipment and has its limitations and drawbacks. It is well known that suboptimal freezing speed during the cryo-fixation causes disruptions to the soft hydrated material due to the water ice crystallization [45]. Therefore, the choice of a particular cryo-fixation technique is crucial to obtain unperturbed structure of the frozen specimen.

**Figure 3:**
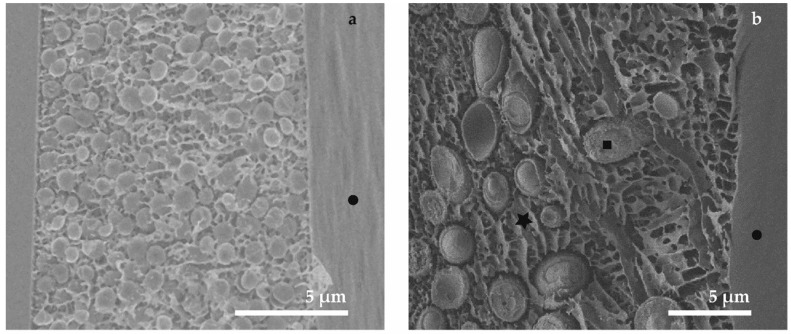

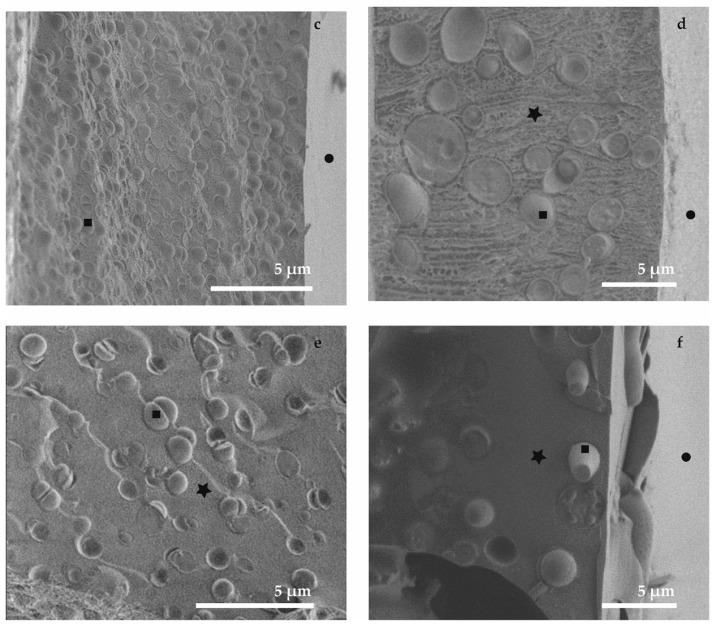
SEM micrographs showing the comparison of preparation protocols applied to microbial biofilms (in the left column *S. epidermidis* and in the right column *C. parapsilosis*): (**a**,**b**) plunge freezing into nitrogen slush; (**c**,**d**) plunge freezing into liquid ethane; (**e**,**f**) freezing by HPF. Marks: stars—fully hydrated ECM, squares—microbes, dots—surface of biofilm layer; measurement parameters 1 to 2 keV and 6–8 mm.

The comparison of the freezing methods for yeast and bacterial biofilms is the main aim of this section of our study. The perpendicular freeze-fracture of the samples (24 h old biofilms of *Staphylococcus epidermidis* and *Candida parapsilosis*) frozen by plunging into nitrogen slush are shown in Figure 3a,b.

**Figure 4:**
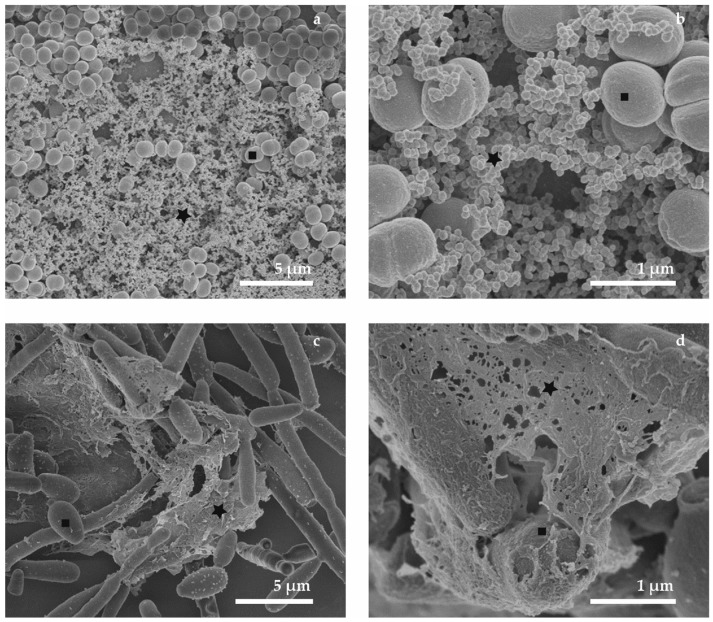
SEM micrographs show the comparison of the bacterial and yeast biofilm structure after the combined sample preparation: (**a**,**b**) *S. epidermidis*; (**c**,**d**) *C. parapsilosis*. In the left column, images are displayed in a lower magnification, while the details are shown in the right column; measurement parameters: 2 keV, WD 6 mm.

The inner structure of the microorganisms (Marks—squares in Figure 4) seems to be sufficiently preserved in the range of magnification around 5000×. The thickness of the biofilm layer is approximately 10 µm. The structure of the ECM containing the extracellular biopolymers and the cultivation medium has a sponge-like character. We consider this structure to represent a micro-segregation (±0.5 µm) caused by ice crystal growing during the slow freezing process. The method based on plunging the sample into liquid ethane should be appropriate for freezing a sample with thickness up to 10 µm [45]. In our micrographs the final structures after fixation by plunging into liquid ethane look very similar to previous experiments, probably due to the existence of a cultivation substrate such as described in literature [76]. Nevertheless, the use of sapphire discs as a cultivation substrate for plunge-freezing seems to be more suitable from the perspective of their thermal conductivity. However, several experiments show that the plunge freezing fixation of cells cultivated on sapphire discs lead to ice crystal segregation as well [77]; this result also corresponds to our experiments. Noticeably better preservation of the whole microbial biofilm, including the extracellular matrix, is clearly visible in Figure 3e,f (marks—stars). High-pressure freezing ranks among the cutting-edge sample preparation techniques for cryo-SEM. Besides the fast cooling rate, a positive influence is apparently brought by the use of sapphire discs with better thermal conductivity compared to other substrates. The structure of ECM (Figure 3e,f, marks—stars) is very smooth within the magnification used and we were able to detect only minimal disruptions due to the ice crystallization. Moreover, it is possible to recognize the denser parts of the biofilm that can be observed in the surroundings of the microbial cells [34].

Cryo-methods in SEM are capable of providing information about the ultrastructure of the microbial biofilm interior, assuming that freeze-fracturing is applied. It is possible to visualize the areas where microbes adhere to the surface of the cultivation substrate and the contact fields between individual microbial cells. On the other hand, examination of the biofilm surface (Figure 3, marks—circles) could not be performed, because the biofilm layer was covered by frozen liquid (the cultivation medium with a content of EPS).

### 3.3. Combined Preparation—Chemical and Cryo-Methods (M6)

Alternative sample preparation protocol is the combination of chemical fixation by solution of GA and OsO_4_ and dehydration by the ethanol series such as in the case of biological sample preparation for conventional SEM techniques by the standard chemical method. When the total water content was replaced by 100% ethanol, the samples in our experiments were frozen by plunging into the nitrogen slush, and transferred to the cryo-preparation chamber where they were sublimated overnight until they were completely dry. The sublimation process started at −140 °C, then temperature increased to −80 °C with a speed of heating 4 °C/min for 6 h and finally samples were left to warm up to the room temperature spontaneously. Additional coating (4 nm, carbon) allowed a production of high-magnification micrographs of our biofilm samples. From our results it can be concluded that the M6 protocol is the most favorable means of biofilm preparation with the aim of surface visualization. The micrographs in Figure 4 show the spatial distribution of microbes and ECM similar to conventional preparation for room-temperature SEM. The ECM (Figure 4, marks—stars) of the bacterial biofilm seems to have an aggregation character which can arise from the chemical treatment during the preparation. The fiber-like structure of the extracellular matrix that covered and interconnected the yeasts bodies looks very similar to the ECM in samples prepared by CPD, but biofilm samples were washed away to a lesser extent due to the absence of chemical drying or CPD. We were not able to detect any evidence of a gel-like matrix covering the microbial cells as described in many publications [1,24,78]. This is to be expected, because the whole freeze-drying procedure was applied in this method. We found that the visualized structures are the remnants of the cultivation medium and the condensed matrix components. The limitations of the six different preparation techniques for SEM on bacterial and yeast biofilms (M1–M6), the benefits and influence on the biofilm structure are summarized in Table 1. 

### 3.4. Analysis of the Yeast and Bacterial Biofilms by Raman Spectroscopy

**Figure 5:**
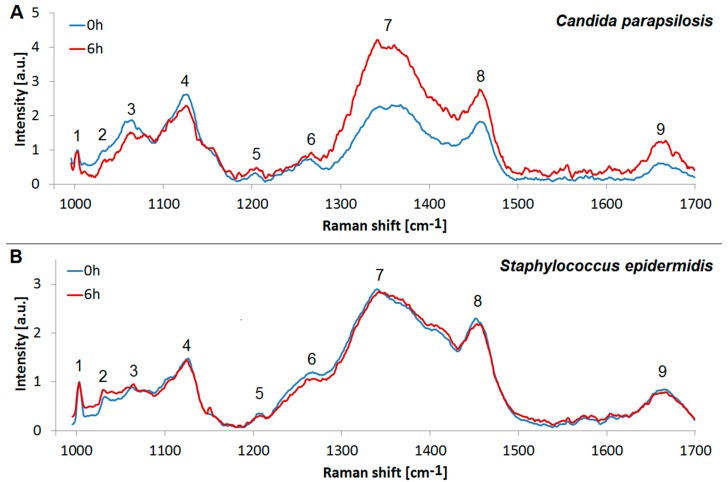
Raman spectra of biofilms. (**A**): *Candida parapsilosis*; (**B**): *Staphylococcus epidermidis*. Comparison of the freshly inoculated substrates containing no ECM (0 h, blue) with 6 h old biofilm, showing the start of the ECM production (6 h, red). The Raman peaks associated with biomolecules are numbered, see Table 2 for the assignments. The spectra were averaged from six separate measurements.

**Figure 6:**
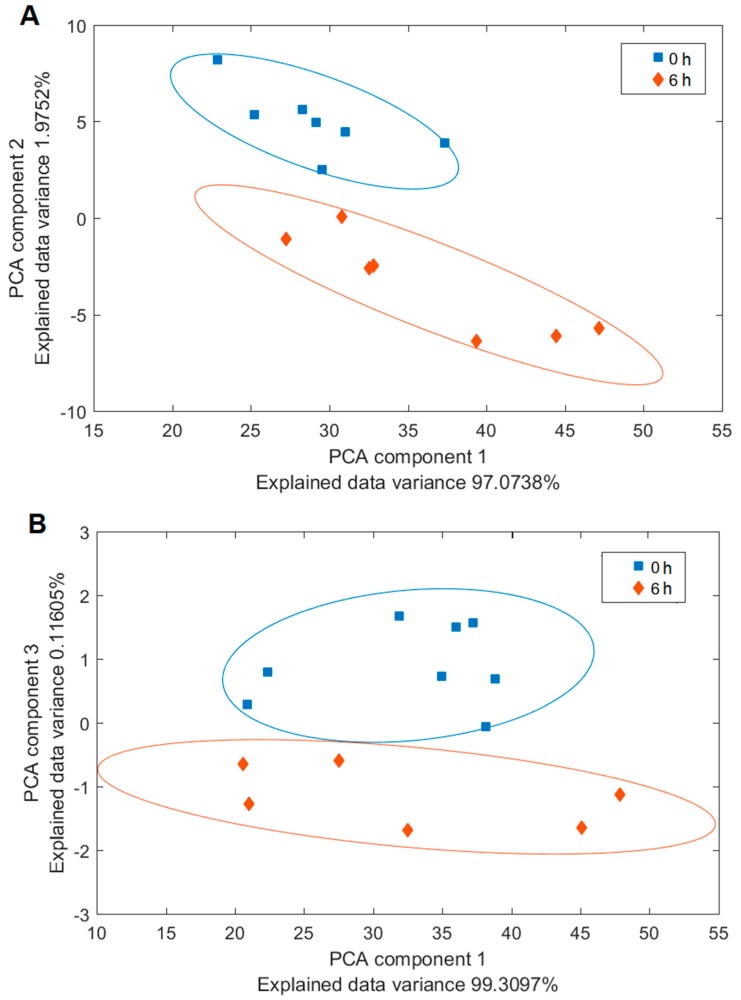
PCA plots for the two species (**A**) *Candida parapsilosis* and (**B**) *Staphylococcus epidermidis*. The clusters of spectra are associated with the two incubation times. Blue squares/0 h—initial cultures with no ECM; red diamonds/6 h—6 h old cultures with starting ECM formation.

**Figure 7:**
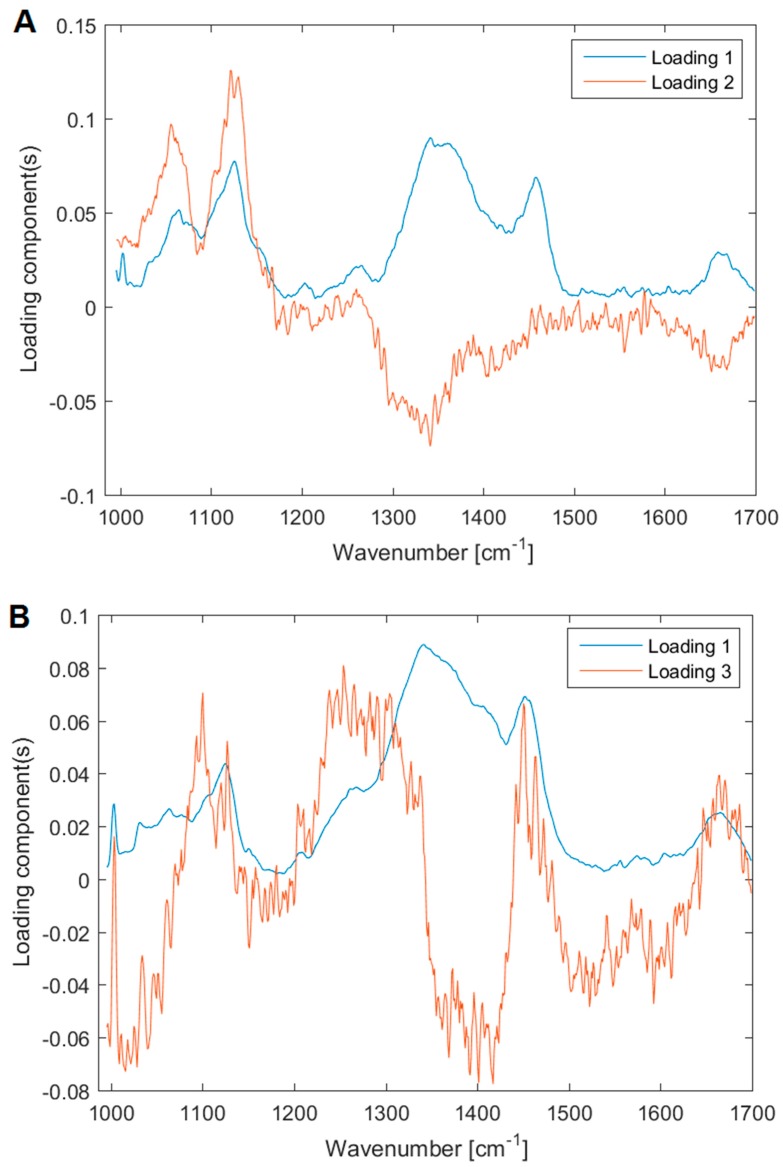
PCA loadings for the two species (**A**) *Candida parapsilosis* and (**B**) *Staphylococcus epidermidis*. The main differences between the cells that did not yet formed the biofilm and the ones that already produce it are presented mainly in the quantity of the generated proteins, sugars and lipids.

We assessed the chemical composition of the biofilms using Raman microspectroscopy. Our central objective was to identify the main components of the biofilm. We compared the Raman spectra from the freshly inoculated substrate, containing only the microbial cells, but no ECM, with relatively fresh (6 h old) biofilm, containing a definite proportion of the ECM, see Figure 5. To this end, we assigned the individual peaks of the measured spectra, see Table 2, and we employed PCA to pinpoint the differences in the chemical composition, see Figure 6. The associated PCA loadings reveal the essential changes associated with the ECM production, see Figure 7. We found the main difference between the freshly inoculated substrate and 6 h old biofilm is in the production of proteins, sugars, and lipids (peaks 6, 7, and 8 on Figure 5). This stems from the generation of various polysaccharides, proteoglycans, lipopolysaccharides and lipoproteins as the basis of the ECM.

While the differences in the spectra of *Candida parapsilosis* at 0 h and 6 h are very pronounced, especially in the region 1340–1360 cm^−1^ and 1456 cm^−1^, *Staphylococcus epidermidis* spectra at 0 h and 6 h show only marginal differences (Figure 5), although they are clearly visible with the aid of the PCA analysis (Figure 6 and Figure 7). This discrepancy is probably caused by the differences in the optimal conditions for biofilm production by the two studied species. *Candida parapsilosis*, which is eukaryotic, may have developed more complex strategies to react to suboptimal or stressful conditions in general, including the ability to generate biofilm more quickly and efficiently, compared to *Staphylococcus epidermidis*, in which the biofilm production may be linked only to a specific set of growth conditions. We conclude that while we were able to stimulate the biofilm growth in *Candida parapsilosis*, in the Raman experiments we did not meet the optimal conditions for biofilm production in *Staphylococcus epidermidis*. However, it is obvious from the SEM observations, that given enough time, the biofilm growth around the *Staphylococcus epidermidis* cells is abundant. 

The chemical changes associated with the formation of biofilm can be easily observed in the PCA loadings in Figure 7. While the *Candida parapsilosis* biofilm appears to be composed mainly of fungal exopolysaccharides that lack nitrogen in their structure (peaks at 1340–1360 cm^−1^) [60,79], the biofilm of *Staphylococcus epidermidis* is relatively richer in nitrogen containing polysaccharides such as poly-*N*-acetylglucosamine (peaks around 1400 cm^−1^) [60,80]. The ECM of *Candida parapsilosis* is composed of several groups of chemical components, including presumably the lipoproteins and proteoglycans. In contrast, the ECM of *Staphylococcus epidermidis* appears to be more chemically uniform. This uniformity apparently stems from the relative simplicity of the bacterial (prokaryotic) metabolism compared to the eukaryotic metabolism of *Candida parapsilosis*.

## 4. Conclusions

In order to study the microbial biofilm structure of *Candida parapsilosis* and *Staphylococcus epidermidis* we have investigated different sample preparation techniques for SEM. The effect of sample preparation for conventional SEM allowing surface imaging at room temperature was compared with cryo-SEM techniques employing plunging into various liquid cryogens and HPF. For the cryo-SEM imaging of the biofilm inner structure we have selected the freeze-fracturing technique. We made a comparison of applied techniques for microbial biofilm studies which indeed showed different influences on the final structure of the biofilm. Based on our findings the best candidate for biofilm evaluation can be selected.

We showed that a combination of Raman spectroscopy with selected SEM techniques can provide a deeper insight into the chemistry and composition of biofilms. Such studies involving the influence of variations in the amount of extracellular material during the different stages of biofilm growth are currently under way in our laboratories, making use of a combination of SEM and Raman spectroscopy. We believe that the detailed view of the biofilm structure and composition can advance the better understanding of biofilm structures.

## Figures and Tables

**Table 1:** Comparison of benefits and limitations of sample preparation protocols for SEM (Methods M1–M6) and their influence on biofilm structure as shown in schematic drawings. Our best candidates for sample preparation techniques are labelled green.

M	Advantage	Disadvantage	Schema
**M1—air-drying**	Speed of sample preparation Simplicity Repeatability of measurement in SEM at room temperature Suitable for **surface imaging**	The loss of the 3D structure Deformation of microbial biofilm Deformation of ECM The possibility of imaging only the sample surface (not interior)	Deformation of biofilm 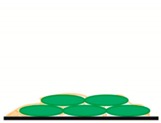
**M2—conventional chemical preparation**	Repeatability of measurement in SEM at room temperature The 3D structure is preserved. Suitable for **surface imaging**	Long-term procedure Damage of soft biofilm sample due to multi-steps washing Artefacts with chemicals treatment (the change of gel-like ECM into fiber structures) The sample surface imaging	Biofilm is washed out 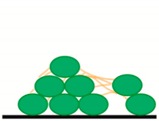
**M3—plunging LN_2_; cryo-SEM**	Speed of sample preparation The 3D structure of microbial cells is preserved Possibility of biofilm **interior imaging** (used freeze-fracturing technique also suitable for M3–M5)	Artefacts with freezing procedure Freezing is sufficient for very thin samples Limitation for surface imaging because of water content in biofilm samples	“Large” ice crystals 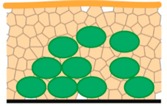
**M4—plung. Ethane; cryo-SEM**	Speed of sample preparation The 3D structure of microbial cells is preserved Possibility of biofilm **interior imaging**	Artifacts with freezing procedure (smaller ice crystals inside biofilm than by M3) Freezing of thin samples Limitation in surface imaging because of water content in biofilm samples	“Small” ice crystals 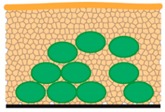
**M5—HPF freezing and cryo-SEM**	Speed of sample preparation 3D structure of microbial cells/ECM is nicely preserved The best freezing technique for samples with thickness up to 200 µm (exp. tested) Biofilm **interior imaging**	Limitation in surface imaging (water content in biofilm) Limitations connected with HPF machine–cultivation substrate (sapphire discs for freeze fracturing; Al or Cu-gold discs)	**Optimal** prep. of biofilm for **interior imaging** 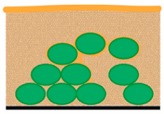
**M6—Combined preparation**	Speed of sample preparation 3D structure of biofilm Biofilm **surface imaging** Repeatability of measurement in SEM at room temperature after freeze-drying Less washed out biofilm	Artifacts from chemical fixation (the change of gel-like ECM) The imaging of sample surface	**Applicable** prep. of chemically fixed biofilm for **surface imaging** 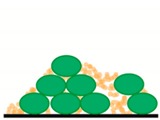

**Table 2:** Assignments of Raman peaks of *C. parapsilosis* and *S.epidermidis* biofilms [51].

No.	Wavenumber [cm^−1^]	Peaks Assignment
1	1002	Symmetric-ring breathing of Phe
2	1033	C-H in-plane stretch of Phe
3	1065	C-C stretch of lipids
4	1125	C-N stretch of proteins
5	1205	Proteins
6	1267	Lipids, Amide III
7	1340–1360	Proteins, Carbohydrates
8	1456	CH_2_ scissoring, Lipids
9	1660	Amide I, Lipids
